# Effect of smoking cessation on the likelihood of pancreatitis and pancreatic cancer

**DOI:** 10.18332/tid/190635

**Published:** 2024-07-12

**Authors:** Xiao Han, Zouhua Xu, Dongmei Ma, Zhi Ling, Xiaowu Dong, Xuebing Yan, Yong Chen, Guotao Lu, Xudong Yin, Hongwei Xu

**Affiliations:** 1 Department of Oncology, Affiliated Hospital of Yangzhou University, Yangzhou University, Yangzhou, China; 2 Yangzhou Key Laboratory of Pancreatic Diseases, Affiliated Hospital of Yangzhou University, Yangzhou University, Yangzhou, China; 3 Kunshan Hospital of Traditional Chinese Medicine, Kunshan Affiliated Hospital of Yangzhou University, Yangzhou University, Kunshan, China; 4 Pancreatic Center, Affiliated Hospital of Yangzhou University, Yangzhou University, Yangzhou, China

**Keywords:** smoking, smoking cessation, pancreatitis, pancreatic cancer

## Abstract

**INTRODUCTION:**

Tobacco smoking is a major risk factor for various diseases worldwide, including pancreatic exocrine diseases such as pancreatitis and pancreatic cancer (PC). Currently, few studies have examined the impact of smoking cessation on the likelihood of common pancreatic exocrine diseases. This study sought to determine whether smoking cessation would reduce pancreatitis and PC morbidity.

**METHODS:**

This cohort study used data from the UK Biobank (UKB) to examine the association between smoking status and the likelihood of pancreatitis and PC among 492855 participants. The subjects were divided into never smokers, ex-smokers, and current smokers. Using a multivariate-adjusted binary logistic regression model, we analyzed the relationship between different smoking conditions and the likelihood of pancreatitis and PC. Further, we studied the impact of smoking cessation on pancreatitis and PC compared with current smoking.

**RESULTS:**

After adjusting for potential confounders, current smokers had higher odds for acute pancreatitis (AP) (AOR=1.38; 95% CI: 1.18–1.61), chronic pancreatitis (CP) (AOR=3.29; 95% CI: 2.35–4.62) and PC (AOR=1.72; 95% CI: 1.42–2.09). People who quit smoking had comparable odds for the diseases as those who never smoked. Compared with current smokers, ex-smokers had reduced odds for AP (AOR=0.76; 95% CI: 0.64–0.89), CP (AOR=0.31; 95% CI: 0.21–0.46), and PC (AOR=0.62; 95% CI: 0.50–0.76). Subgroup analysis revealed reduced odds for these pancreatic diseases in males and females.

**CONCLUSIONS:**

Smokers have an increased odds for pancreatitis and pancreatic cancer. Moreover, smoking cessation can significantly reduce the odds for acute pancreatitis, chronic pancreatitis and pancreatic cancer.

## INTRODUCTION

Smoking is associated with many diseases worldwide. Carbonyl, N-nitrosamines, polycyclic aromatic hydrocarbons, and benzene are toxic components of tobacco smoke^1,2^. Globally, more than 1 billion adults are currently engaged in smoking behavior, with the majority expressing a desire to quit; however, only a limited number achieve successful cessation. Smoking is also a major contributor to the global disease burden^3^. Most deaths from smoking are attributable to chronic obstructive pulmonary disease, ischemic heart disease, lung cancer, and stroke^4^. Studies have found that smoking cessation before the age of 40 years can reduce the odds for smoking-related death by about 90%^5^.

Numerous studies have shown a strong correlation between smoking and pancreatic damage^6^. The pancreas is the second largest digestive gland in the human body, consisting of the endocrine part that mainly secretes insulin and glucagon and the exocrine part that secretes pancreatic juice. Malfunctioning pancreatic secretion leads to pancreatic exocrine diseases: acute pancreatitis (AP), chronic pancreatitis (CP), and pancreatic cancer (PC) are the most common^6^. The global incidence of AP is 5–80 cases per 100000 people^7^, consistent with the incidence of CP, increasing annually^8^. PC, similar to pancreatitis, is expected to reach 18.6 cases per 100000 people worldwide by 2050, with an annual growth rate of about 1.1%^9^. Currently, the treatment of AP, CP, and PC is complex, with limited efficacy^10-12^, high recurrence rate, and poor prognosis, posing a serious threat to human health^13^.

Studies have shown that smoking is associated with AP, CP, and PC onset^14-17^. In this study, we aimed to assess the odds for developing pancreatic disease among smokers and non-smokers, and those with or without smoking cessation.

## METHODS

### Study design and subjects

The UK Bio-sample database is the world’s largest biomedical sample database, tracking sample data from around 500000 participants recruited between 2006 and 2020, aged 40–69 years at the time of recruitment^18^. With their consent, they regularly provide detailed information about blood, urine, and saliva samples and their lifestyle at clinical centers across England, Wales, and Scotland. This is then linked to their health-related records to provide a deeper understanding of how individuals experience diseases^19^. The study aims to observe if there is a difference between smoking cessation and non-smoking cessation in terms of the odds for pancreatic disease. The subjects were divided into smokers and non-smokers, ex-smokers and non-ex-smokers.

The project was approved by the British National Research Ethics Committee North West-Haydock. All participants provided written informed consent before participating in the study.

### Inclusion and exclusion criteria

The inclusion criteria were all participants in the UKB.

Exclusion criteria were: 1) loss to follow-up; 2) lack of data such as sex, age, body mass index (BMI), Townsend Deprivation Index (TDI), smoking, and alcohol consumption; and 3) those with a history of exocrine pancreatic disease before participating in the assessment.

### Baseline data

Sex, age, race, smoking, and alcohol consumption were self-reported at baseline. BMI was calculated using height and weight, measured by the assessment center. The TDI is a composite score based on four variables: unemployment, overcrowded households, non-car, and non-home ownership. Higher scores on the index indicate higher deprivation levels^20^. A history of diabetes mellitus (DM) and high blood pressure (HBP) was obtained through self-reports and hospital databases.

### Outcome evaluation

According to the 9th and 10th International Classification of Diseases (ICD) issued by the World Health Organization (WHO), we extracted the disease codes of all samples from the UKB database. The outcome event of the study was pancreatitis or PC diagnosis, determined by whether the age at the time of diagnosis was greater than the age at the time of recruitment and confirmed by medical record review. Specific disease codes are given in Supplementary file Table 1.

**Table 1:** Characteristics of subjects by different smoking status, to examine the association between smoking status and the likelihood of pancreatitis and pancreatic cancer, UK Biobank, 2006–2020 (N=492855)

*Characteristics*	*Never smokers (N=270092) n (%)*	*Ex-smokers (N=170885) n (%)*	*Current smokers (N=51878) n (%)*	*χ^2^/Z*	*p*
**Sex**				5718.44	<0.001
Female	160095 (59.3)	84459 (49.4)	23938 (46.1)		
Male	109997 (40.7)	86426 (50.6)	27940 (53.9)		
**Age** (years)	57 (50-63)	60 (53-65)	55 (48-62)	9157.4	<0.001
<60	158124 (58.5)	78053 (45.7)	33359 (64.3)		
≥60	111968 (41.5)	92832 (54.3)	18519 (35.7)		
**TDI** (Q1-Q3)	-2 (-4–0)	-2 (-4–1)	0 (-3–3)	11588.98	<0.001
**Race**				1031.57	<0.001
White	245450 (90.9)	155748 (91.1)	45832 (88.3)		
Mixed	10031 (3.7)	5996 (3.5)	2360 (4.5)		
Asian	8162 (3.0)	6595 (3.9)	2341 (4.5)		
Black	1714 (0.6)	648 (0.4)	416 (0.8)		
Other	4735 (1.8)	1898 (1.1)	929 (1.8)		
**BMI** (kg/m^2^)	26 (24–30)	27 (25–30)	26 (24–30)	3375.05	<0.001
<25	83137 (30.8)	40362 (23.6)	16299 (31.4)		
25–29.99	117787 (43.6)	77468 (45.3)	22324 (43.0)		
≥30	69168 (25.6)	53055 (31.0)	13255 (25.6)		
**Drinking**	244587 (90.6)	161345 (94.4)	47757 (92.1)	2132.21	<0.001
**HBP**	71204 (26.4)	60290 (35.3)	15915 (30.7)	3987.20	<0.001
**DM**	18670 (6.9)	17538 (10.3)	5447 (10.5)	1832.86	<0.001
**AP**	1249 (0.5)	1021 (0.6)	352 (0.7)	59.59	<0.001
**CP**	236 (0.1)	256 (0.1)	178 (0.3)	213.36	<0.001
**PC**	766 (0.3)	630 (0.4)	253 (0.5)	63.47	<0.001

### Statistical methods

The data was cleaned and imported to SPSS25.0 using Excel. The counting data with a normal distribution were represented as mean ± standard deviation, and the ANOVA test or the Kruskal-Walis H test was selected according to the homogeneity of variance test. Data with a non-normal distribution were expressed as median and interquartile range using the Kruskal-Walis H test. Categorical data are presented as percentages, and χ^2^ was calculated and used to calculate p values. The association between smoking, smoking cessation and pancreatitis, and PC was assessed by binary logistic regression analysis by adjusting for confounding factors such as sex, age, race, BMI, TDI, drinking, DM, and HBP and expressed as p values and adjusted odds ratios (AORs), with confidence intervals (CI) of 95%. We used subgroup analysis to identify potential effect modifiers and calculated the p-value for interaction (pinteraction).

## RESULTS

### Participants

**Figure 1:**
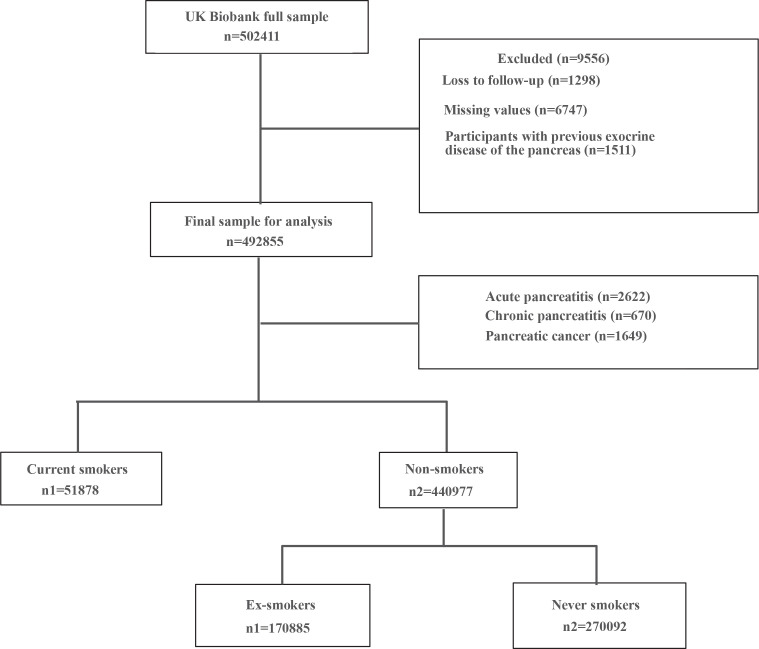
Flowchart for data filtering, UK Biobank, 2006–2020 (N=502411)

Overall, 502411 participants were included within the time frame of the present study. Of those, 492855 met the eligibility criteria of the study (Figure 1) comprising 270092 never smokers, 170885 ex-smokers, and 51878 current smokers. As shown in Table 1, there were significant differences in the distribution of people in terms of sex, age, race, BMI, TDI, drinking status, HBP, and DM. AP, CP, and PC likelihood varied with smoking status, and the differences were statistically significant (p<0.001).

### Outcome data

*Association of smoking status with the likelihood of AP, CP, and PC*

The odds for AP, CP, and PC for never smokers, ex-smokers, and current smokers are shown in Table 2. The adjusted odds for AP (AOR=1.38; 95% CI: 1.18–1.61), CP (AOR=3.29; 95% CI: 2.35–4.62), and PC (AOR=1.72; 95% CI: 1.42–2.09) were significantly higher in current smokers than in the never smokers. However, the odds for the diseases in people who quit smoking were comparable to those who never smoked.

**Table 2:** Univariate and multivariate logistic regression model explaining self-reported smoking status with the likelihood of acute pancreatitis, chronic pancreatitis and pancreatic cancer, UK Biobank, 2006–2020 (N=492855)

*Variable*	*Smoking status*	*Model 1*		*Model 2*		*Model 3*	
		*OR (95% CI)*	*p*	*AOR (95% CI)*	*p*	*AOR (95% CI)*	*p*
**AP**	Never smoker ®	1	<0.001	1	<0.001	1	<0.001
	Ex-smoker	1.29 (1.19–1.41)	<0.001	1.15 (1.06–1.25)	0.001	1.02 (0.91–1.14)	0.753
	Current smoker	1.47 (1.31–1.66)	<0.001	1.40 (1.24–1.58)	<0.001	1.38 (1.18–1.61)	<0.001
**CP**	Never smoker ®	1	<0.001	1	<0.001	1	<0.001
	Ex-smoker	1.72 (1.44–2.05)	<0.001	1.46 (1.22–1.75)	<0.001	0.97 (0.68–1.37)	0.844
	Current smoker	3.94 (3.24–4.78)	<0.001	3.25 (2.66–3.97)	<0.001	3.29 (2.35–4.62)	<0.001
**PC**	Never smoker ®	1	<0.001	1	<0.001	1	<0.001
	Ex-smoker	1.30 (1.17–1.45)	<0.001	1.09 (0.98–1.21)	0.128	0.99 (0.85–1.14)	0.876
	Current smoker	1.72 (1.49–1.99)	<0.001	1.75 (1.51–2.02)	<0.001	1.72 (1.42–2.09)	<0.001

Model 1: not adjusted. AOR: adjusted odds ratio. Model 2: adjusted for sex, age, race, BMI, TDI, drinking. Model 3: adjusted for sex, age, race, BMI, TDI, drinking, DM and HBP; while AP adjusted for CP and PC, CP adjusted for AP and PC, PC adjusted for AP and CP. ® Reference category.

*Association of smoking cessation with the likelihood of AP, CP, and PC*

Group differences are shown in Table 3. Following adjustment for relevant confounding factors, compared with current smokers, the odds for AP decreased by about 25% in people who had quit smoking (AOR=0.76; 95% CI: 0.64–0.89; p=0.001), CP decreased by about 70% (AOR=0.31; 95% CI: 0.21–0.46; p<0.001), and PC decreased by about 40% (AOR=0.62; 95% CI: 0.50–0.76; p<0.001) in Model 3.

**Table 3:** Univariate and multivariate logistic regression model explaining smoking cessation with the likelihood of acute pancreatitis, chronic pancreatitis and pancreatic cancer, UK Biobank, 2006–2020 (N=492855)

*Variable*	*Model 1*		*Model 2*		*Model 3*	
	*OR (95% CI)*	*p*	*AOR (95% CI)*	*p*	*AOR (95% CI)*	*p*
**AP**	0.88 (0.78–0.99)	0.039	0.84 (0.74–0.95)	0.007	0.76 (0.64–0.89)	0.001
**CP**	0.44 (0.36–0.53)	<0.001	0.46 (0.38–0.57)	<0.001	0.31 (0.21–0.46)	<0.001
**PC**	0.76 (0.65–0.87)	<0.001	0.65 (0.56–0.76)	<0.001	0.62 (0.50–0.76)	<0.001

Model 1: not adjusted. AOR: adjusted odds ratio. Model 2: adjusted for sex, age, race, BMI, TDI, drinking. Model 3: adjusted for sex, age, race, BMI, TDI, drinking, DM and HBP; while AP adjusted for CP and PC, CP adjusted for AP and PC, PC adjusted for AP and CP.

### Subgroup analysis

As shown in Supplementary file Table 2, after grouping by sex, the odds for AP, CP, and PC were lower in males and females after smoking cessation in Model 3. There was no differential association of smoking status or cessation with the pancreatic diseases by sex (AP: p_interaction_=0.845; CP: p_interaction_=0.642; and PC: p_interaction_=0.958).

## DISCUSSION

Currently, the clinical research on the effect of smoking cessation on pancreatitis and PC is insufficient. Using a large UKB database containing nearly 500000 samples, smoking was used as a single exposure factor for the analysis of clinical data in this study, proving smoking to be a factor for AP, CP, and PC. On this basis, we further studied the impact of smoking cessation on pancreatitis and PC, and we confirmed that the odds for both decreased after smoking cessation, by constantly adjusting possible confounding factors.

It is well known that smoking is a factor for pancreatitis, mainly by inducing oxidative stress, reducing bicarbonate secretion, and stimulating pancreatic fibrosis^21^. Szentesi et al.^6^ and Aune et al.^22^ demonstrated a dose-dependent correlation between smoking and pancreatic tissue damage and the likelihood of pancreatitis in the clinical setting. Smoking is also an environmental factor for PC. Pancreatic ductal adenocarcinoma (PDAC) is the most common pathological type of PC, primarily driven by the K-Ras oncogene^23^. Tobacco smoke stimulates inflammatory cell infiltration: interleukin (IL)-6, IL-11, and tumor necrosis factor (TNF)-α, to produce an inflammatory response^24,25^, initiating and accelerating the progression of PC by working with Kirsten rat sarcoma viral oncogene (KRAS)^26^. The study found that the odds for AP (OR=1.36; 95% CI: 1.17–1.58), CP (OR=3.30; 95% CI: 2.43–4.50) and PC (OR=1.73; 95% CI: 1.44–2.08) were significantly higher in smokers than in non-smokers, which further verified the above view.

However, whether quitting smoking reduces the odds for pancreatitis and PC is debatable. Sadr-Azodi et al.^27^ found that the odds for AP after 20 years of smoking cessation can be reduced to baseline levels in never smokers. A prospective study from the Chinese Chronic Disease Biological Database also showed that the odds for AP were decreased in people who quit smoking^14^. Majumder et al.^28^ proposed in a meta-analysis that the odds for AP among ex-smokers remained significantly higher than among non-smokers (HR=1.63; 95% CI: 1.18–2.27). Moreover, differences in outcomes due to insufficient duration of smoking cessation could not be ruled out. There are few clinical studies on the effect of smoking cessation on the likelihood of CP, concluding that compared with current smokers, the odds for AP and CP were significantly reduced in people who quit smoking regardless of sex.

A 2019 Japanese review of 10 cohort studies on the association between smoking and the incidence of PC found that the odds for PC decreased in men after five years of smoking cessation but not in women^29^. This study reported that the odds for PC in former smokers were significantly lower than those in current smokers, with the odds reduced by about 40% for males and about 35% for females, confirming the positive benefits of smoking cessation. Molina-Montes et al.^30^ also reported reduced PC odds after smoking cessation. It is estimated that quitting smoking can reduce the likelihood of PC-related death by about 25%^31^.

### Strengths and limitations

The results of this study are relatively reliable because they are based on a cohort study of approximately 500000 large samples, and the confounding factors associated with pancreatic exocrine disease, such as sex, age, alcohol consumption, BMI, hypertension, diabetes, etc., have been adjusted. In addition, we found that sex differences in smoking cessation do not play a protective role in pancreatic disease.

However, there are some limitations in this study. First, due to the scarcity of data sources, the ethnic distribution was uneven, and the influence of residual confounding factors cannot be completely ruled out. Second, the baseline data used in this study cannot reflect the dynamic changes of individuals. For example, at the time of enrollment, some people were in a smoking cessation state, and it is unknown whether they smoked again during the observation period. Third, if data regarding the number of years of smoking cessation were available, this would have strengthened the study. Further, the results will be more convincing if smoking cessation-related biochemical indicators were employed. Fourth, the number of cases of AP, CP, and PC were insufficient because of the limited follow-up time of the UKB queue.

## CONCLUSIONS

We confirmed that smoking leads to increased odds for pancreatitis and PC, through a systematic analysis of prospective data from UKB, and further analysis showed that smoking cessation reduces the odds for AP, CP, and PC. Therefore, it is necessary to provide health education among smokers and persuade them to quit smoking early.

## Supplementary Material



## Data Availability

The datasets were derived from sources in the public domain: UK Biobank under application number 69476.

## References

[1] Willett J, Achenbach S, Pinto FJ, Poppas A, Elkind MSV. The tobacco endgame-eradicating a worsening epidemic. Eur Heart J. 2021;42(32):3044-3048. doi:10.1093/eurheartj/ehab245 34418055 PMC8380060

[2] Münzel T, Hahad O, Kuntic M, Keaney JF, Deanfield JE, Daiber A. Effects of tobacco cigarettes, e-cigarettes, and waterpipe smoking on endothelial function and clinical outcomes. Eur Heart J. 2020;41(41):4057-4070. doi:10.1093/eurheartj/ehaa460 32585699 PMC7454514

[3] Zhou M, Wang H, Zeng X, et al. Mortality, morbidity, and risk factors in China and its provinces, 1990-2017: a systematic analysis for the Global Burden of Disease Study 2017. Lancet. 2019;394(10204):1145-1158. doi:10.1016/S0140-6736(19)30427-1 31248666 PMC6891889

[4] Wen H, Xie C, Wang F, Wu Y, Yu C. Trends in disease burden attributable to tobacco in China, 1990-2017: findings from the Global Burden of Disease Study 2017. Front Public Health. 2020;8:237. doi:10.3389/fpubh.2020.00237 32766191 PMC7381278

[5] Toll BA, Rojewski AM, Duncan LR, et al. “Quitting smoking will benefit your health”: the evolution of clinician messaging to encourage tobacco cessation. Clin Cancer Res. 2014;20(2):301-309. doi:10.1158/1078-0432.CCR-13-2261 24436474 PMC3927319

[6] Szentesi A, Farkas N, Sipos Z, et al. Alcohol consumption and smoking dose-dependently and synergistically worsen local pancreas damage. Gut. 2022;71(12):2601-2602. doi:10.1136/gutjnl-2021-326853 35046088 PMC9664132

[7] Leppäniemi A, Tolonen M, Tarasconi A, et al. 2019 WSES guidelines for the management of severe acute pancreatitis. World J Emerg Surg. 2019;14:27. doi:10.1186/s13017-019-0247-0 31210778 PMC6567462

[8] Beyer G, Habtezion A, Werner J, Lerch MM, Mayerle J. Chronic pancreatitis. Lancet. 2020;396(10249):499-512. doi:10.1016/S0140-6736(20)31318-0 32798493

[9] Hu JX, Zhao CF, Chen WB, et al. Pancreatic cancer: a review of epidemiology, trend, and risk factors. World J Gastroenterol. 2021;27(27):4298-4321. doi:10.3748/wjg.v27.i27.4298 34366606 PMC8316912

[10] Zhou W, Dong S, Chen Z, Li X, Jiang W. New challenges for microRNAs in acute pancreatitis: progress and treatment. J Transl Med. 2022;20(1):192. doi:10.1186/s12967-022-03338-2 35509084 PMC9066850

[11] Engjom T, Waage A, Hoem D, Kvamme JM, Hauge T, Dimcevski G. Kronisk pankreatitt – utredning og behandling. Tidsskr Nor Laegeforen. 2018;138(3):10.4045/tidsskr.17.0341. doi:10.4045/tidsskr.17.0341 29411568

[12] Robatel S, Schenk M. Current limitations and novel perspectives in pancreatic cancer treatment. Cancers (Basel). 2022;14(4):985. doi:10.3390/cancers14040985 35205732 PMC8870068

[13] Han L, Zhao Z, Yang K, et al. Application of exosomes in the diagnosis and treatment of pancreatic diseases. Stem Cell Res Ther. 2022;13(1):153. doi:10.1186/s13287-022-02826-y 35395948 PMC8994331

[14] Pang Y, Kartsonaki C, Turnbull I, et al. Metabolic and lifestyle risk factors for acute pancreatitis in Chinese adults: a prospective cohort study of 0.5 million people. PLoS Med. 2018;15(8):e1002618. doi:10.1371/journal.pmed.1002618 30067849 PMC6070164

[15] Yuan S, Giovannucci EL, Larsson SC. Gallstone disease, diabetes, calcium, triglycerides, smoking and alcohol consumption and pancreatitis risk: Mendelian randomization study. NPJ Genom Med. 2021;6(1):27. doi:10.1038/s41525-021-00189-6 33782414 PMC8007637

[16] Spagnolo DM, Greer PJ, Ohlsen CS, et al. Acute and chronic pancreatitis disease prevalence, classification, and comorbidities: A cohort study of the UK BioBank. Clin Transl Gastroenterol. 2022;13(1):e00455. doi:10.14309/ctg.0000000000000455 35060944 PMC8806365

[17] Huang J, Lok V, Ngai CH, et al. Worldwide burden of, risk factors for, and trends in Pancreatic Cancer. Gastroenterology. 2021;160(3):744-754. doi:10.1053/j.gastro.2020.10.007 33058868

[18] Collins R. What makes UK Biobank special?. Lancet. 2012;379(9822):1173-1174. doi:10.1016/S0140-6736(12)60404-8 22463865

[19] Cornelis MC, Agarwal P, Holland TM, van Dam RM. MIND dietary pattern and its association with cognition and incident dementia in the UK Biobank. Nutrients. 2023;15(1):32. doi:10.3390/nu15010032 PMC9823700 36615690

[20] Ye J, Wen Y, Sun X, et al. Socioeconomic deprivation index is associated with psychiatric disorders: an observational and genome-wide gene-by-environment interaction analysis in the UK Biobank cohort. Biol Psychiatry. 2021;89(9):888-895. doi:10.1016/j.biopsych.2020.11.019 33500177

[21] Sahin-Tóth M, Hegyi P. Smoking and drinking synergize in pancreatitis: multiple hits on multiple targets. Gastroenterology. 2017;153(6):1479-1481. doi:10.1053/j.gastro.2017.10.031 29100845

[22] Aune D, Mahamat-Saleh Y, Norat T, Riboli E. Tobacco smoking and the risk of pancreatitis: a systematic review and meta-analysis of prospective studies. Pancreatology. 2019;19(8):1009-1022. doi:10.1016/j.pan.2019.09.004 31668562

[23] Liu J, Kang R, Tang D. The art of war: Ferroptosis and Pancreatic Cancer. Front Pharmacol. 2021;12:773909. doi:10.3389/fphar.2021.773909 34955844 PMC8702849

[24] Weissman S, Takakura K, Eibl G, Pandol SJ, Saruta M. The diverse involvement of cigarette smoking in pancreatic cancer development and prognosis. Pancreas. 2020;49(5):612-620. doi:10.1097/MPA.0000000000001550 32433397 PMC7249487

[25] Edderkaoui M, Xu S, Chheda C, et al. HDAC3 mediates smoking-induced pancreatic cancer. Oncotarget. 2016;7(7):7747-7760. doi:10.18632/oncotarget.6820 26745602 PMC4884951

[26] Yuan C, Morales-Oyarvide V, Khalaf N, et al. Prediagnostic inflammation and pancreatic cancer survival. J Natl Cancer Inst. 2021;113(9):1186-1193. doi:10.1093/jnci/djab040 33739411 PMC8522350

[27] Sadr-Azodi O, Andrén-Sandberg Å, Orsini N, Wolk A. Cigarette smoking, smoking cessation and acute pancreatitis: a prospective population-based study. Gut. 2012;61(2):262-267. doi:10.1136/gutjnl-2011-300566 21836026

[28] Majumder S, Gierisch JM, Bastian LA. The association of smoking and acute pancreatitis: a systematic review and meta-analysis. Pancreas. 2015;44(4):540-546. doi:10.1097/MPA.0000000000000301 25872130

[29] Koyanagi YN, Ito H, Matsuo K, et al. Smoking and pancreatic cancer incidence: a pooled analysis of 10 population-based cohort studies in Japan. Cancer Epidemiol Biomarkers Prev. 2019;28(8):1370-1378. doi:10.1158/1055-9965.EPI-18-1327 31113869

[30] Molina-Montes E, Van Hoogstraten L, Gomez-Rubio P, et al. Pancreatic cancer risk in relation to lifetime smoking patterns, tobacco type, and dose-response relationships. Cancer Epidemiol Biomarkers Prev. 2020;29(5):1009-1018. doi:10.1158/1055-9965.EPI-19-1027 32051190

[31] Scherübl H. Tobacco smoking and gastrointestinal cancer risk. Visc Med. Jun 2022;38(3):217-222. doi:10.1159/000523668 PMC9209969 35814979

