# Supplementary material for: Effect of smoking cessation on the likelihood of pancreatitis and pancreatic cancer
Source: Tob Induc Dis. 2024 Jul 12;22:10.18332/tid/190635. doi: 10.18332/tid/190635 (PMC11241968; doi:10.18332/tid/190635)
Supplement: Supplementary file 1 [file TID-22-130-s1.pdf]

Supplementary Material

Supplemental Table 1. Detailed coding and classification of the diseases

Diabetes Mellitus	ICD9: 250;<br>ICD10: E10, E11, E12, E13, E14;
Hypertension	ICD9: 401;<br>ICD10: I10, I15, I15.0, I15.1, I15.2, I15.8, I15.9;
Acute pancreatitis	ICD9: 5770;<br>ICD10: K85, K85.0, K85.1, K85.2, K85.3; K85.8, K85.9;
Chronic pancreatitis and other pancreatic diseases	ICD9: 5771;<br>ICD10: K86.0, K86.1, K86.2, K86.3, K86.8, K86.9, K87.1;
Pancreatic cancer	ICD9: 157;<br>ICD10: C25, C25.0, C25.1, C25.2, C25.3, C25.4, C25.7, C25.8, C25.9;

Supplemental Table 2. Univariate and multivariate logistic regression model explaining smoking cessation population of different sexes with the likelihood of AP, CP, and PC, UK Biobank, 2006 –2020 (N= 492855)

Variable	*Sex*	*Model I*			*Model II*			*Model III*		
		OR(95%CI)	P	*p Value for Interaction*	OR(95%CI)	P	*p Value for Interaction*	OR(95%CI)	P	*p Value for Interaction*
AP	female	0.90(0.75-1.09)	0.301	0.790	0.90(0.74-1.10)	0.303	0.521	0.78(0.61-0.99)	0.039	0.845
	male	0.87(0.75-1.02)	0.096		0.80(0.68-0.94)	0.008		0.73(0.58-0.92)	0.008	
CP	female	0.40(0.28-0.56)	<0.001	0.428	0.43(0.30-0.62)	<0.001	0.462	0.30(0.17-0.54)	<0.001	0.642
	male	0.47(0.37-0.59)	<0.001		0.48(0.38-0.61)	<0.001		0.32(0.20-0.53)	<0.001	
PC	female	0.63(0.50-0.79)	<0.001	0.028	0.57(0.45-0.72)	<0.001	0.055	0.64(0.48-0.87)	0.004	0.958
	male	0.88(0.72-1.06)	0.177		0.72(0.59-0.88)	0.001		0.60(0.45-0.80)	<0.001	

Notes: The odds of diseases among current smokers was used as a reference.
Model I: Not adjusted.
Model II: Adjusted for age, race, BMI, TDI, drinking.
Model III: Adjusted for age, race, BMI, TDI, drinking, DM and HBP, while AP adjusted for CP and PC, CP adjusted for AP and PC, PC adjusted for AP and CP.

©2024 Han X. et al.
